# Correlation between clinical features and magnetic resonance imaging findings in lumbar disc prolapse

**DOI:** 10.4103/0019-5413.65148

**Published:** 2010

**Authors:** Aithala P Janardhana, Rajagopal, Sharath Rao, Asha Kamath

**Affiliations:** Department of Orthopaedics, Kasturba Medical College, Manipal University, Manipal, India; 1 Department of Radiology, Kasturba Medical College, Manipal University, Manipal, India; 2 Department of Community Medicine and Statistics, Kasturba Medical College, Manipal University, Manipal, India

**Keywords:** Lumbar disc prolapse, magnetic resonance imaging, clinical correlation

## Abstract

**Background:**

Lumbar disc prolapse is one of the common causes of low back pain seen in the working population. There are contradictorty reports regarding the clinical significance of various magnetic resonance imaging (MRI) findings observed in these patients. The study was conducted to correlate the abnormalities observed on MRI and clinical features of lumbar disc prolapse.

**Materials and Methods:**

119 clinically diagnosed patients with lumbar disc prolapse were included in the study. Clinical evaluation included pain distribution, neurological symptoms and signs. MR evaluation included grades of disc degeneration, type of herniation, neural foramen compromise, nerve root compression, and miscellaneous findings. These MRI findings were tested for inter- and intraobserver variability. The MRI findings were then correlated with clinical symptoms and the level of disc prolapse as well as neurological signs and symptoms. Statistical analysis included the Kappa coefficient, Odd’s ratio, and logistic regression analysis.

**Results:**

There were no significant inter- or intraobserver variations for most of MRI findings (Kappa value more than 0.5) except for type of disc herniation which showed a interobserver variation of 0.46 (Kappa value). The clinical level of pain distribution correlated well with the MRI level (Kappa 0.8), but not all disc bulges produced symptoms. Central bulges and disc protrusions with thecal sac compression were mostly asymptomatic, while centrolateral protrusions and extrusions with neural foramen compromise correlated well with the dermatomal distribution of pain. Root compression observed in MRI did not produce neurological symptoms or deficits in all patients but when deficits were present, they correlated well with the presence of root compression in MRI. Multiple level disc herniations with foramen compromise were strongly associated with the presence of neurological signs.

**Conclusions:**

The presence of centrolateral protrusion or extrusion with gross foramen compromise correlates with clinical signs and symptoms very well, while central bulges and disc protrusions correlate poorly with clinical signs and symptoms. The presence of neural foramen compromise is more important in determining the clinical signs and symptoms while type of disc herniation (bulge, protrusion, or extrusion) correlates poorly with clinical signs and symptoms.

## INTRODUCTION

Lumbar disc prolapse is one of the commonest causes of low back pain in the working population.^1^,^2^ The magnetic resonance imaging (MRI) has provided clinicians with a noninvasive mechanism for viewing lumbar anatomy in great detail.^2^–^7^ Various pathoanatomical changes in lumbar disc prolapse can be visualized in MRI. However, the clinical significance of MRI findings is still controversial. Despite the fact that MRI is done routinely for patients with suspected intervertebral disc prolapse, one is not sure which of the MRI findings are clinically relevant, and have diagnostic as well as prognostic value. Milette *et al*.^5^ found that loss of disc height or abnormal signal intensity is highly predictive of symptomatic tears and the presence of a disc bulge or protrusion does not have additional significance. Beattie *et al*.^8^ found that the presence of disc extrusion and severe nerve root compression at one or multiple sites is strongly associated with distal leg pain. However, Rankine *et al*.^9^ in their study opined that pain drawing is not a good predictor of nerve compression on magnetic resonance imaging with poor correlation. Borenstein and others^3^ clearly opined that MRI findings were not predictive of the development or duration of low back pain and that clinical correlation is essential. So there are questions to be answered. Is MRI really essential in all patients with lumbar disc prolapse? If MRI is done, which of MRI findings will be clinically significant, and which of these findings are important from the management point of view? So we decided to study the correlation between clinical features and MRI findings in lumbar disc prolapse and to know about its significance in decision making for treatment. The present study was conducted to determine the association between abnormalities visible in MRI and patients’ clinical features including pain distribution, neurological signs, and symptoms in lumbar disc prolapse.

## MATERIALS AND METHODS

Patients presenting with clinical features of lower limb radiculopathy to the outpatient department of orthopedics were screened for inclusion in the study. The patients with clinical diagnosis of lumbar disc prolapse were included in the study. Patients who presented with acute onset of symptoms and were having radicular symptoms for the first time were treated with simple bed rest and analgesics for 3 weeks and patients who had completely recovered were excluded from the study. The clinical criteria used are: a) low backache with radiation to the lower limb, b) radicular pain along a specific dermatome, c) nerve root tension signs like straight leg raising test (SLRT) and d) presence of neurological symptoms and signs.

Three of four criteria^7^ should be fulfilled for the diagnosis of lumbar disc prolapse. Patients with two positive criteria, when other causes were ruled out and MRI showed disc prolapse, were also included in the study. All patients were clinically evaluated for pain distribution and presence of neurological symptoms and signs. The dermatomal level of pain distribution was noted. Similarly, the dermatomal level for neurological signs and symptoms was also recorded. The criteria used to determine the dermatomal level are^10^; L3 level: pain or neurological symptoms in the anterior aspect of thigh and knee, L4 level: pain or neurological symptoms in the lower anterior knee or medial aspect of leg and ankle, L5 level: pain or neurological symptoms in the anterolateral aspect of leg and dorsum of foot, S1 level: pain or neurological symptoms in the posterior aspect of leg or sole of foot and, nonspecific: pain in the gluteal region or posterior aspect of thigh or any other patterns which do not fit into any of above category. When the leg pain distribution involves more than one dermatomal level, it was recorded as symptomatic of two levels or more depending on the areas involved.

**Figure 1:**
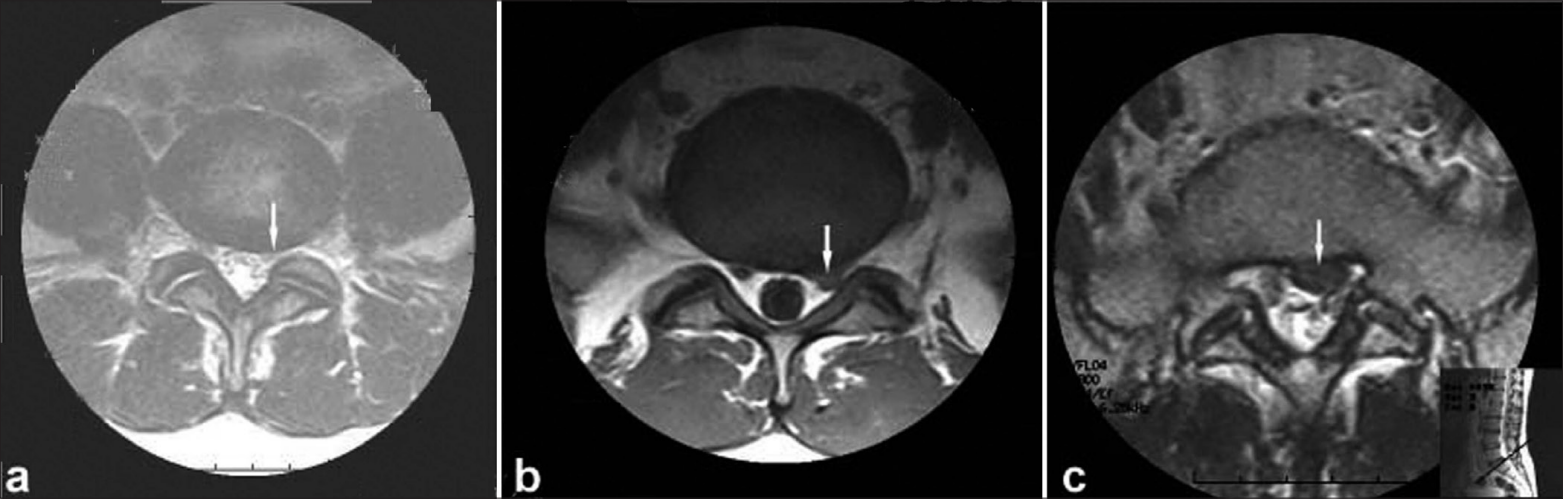
Axial section on MRI depicting classification of disc herniation. (a) Circumferential symmetrical disc extension beyond the interspace (Disc Bulge) (b) Focal or asymmetrical disc extension beyond the interspace with base against the parent disc broader than any other diameter of the protrusion (Disc protrusion) (c) Focal obvious disc extension beyond the interspace with base against the parent disc narrower than the diameter of the extruding material itself or no connection to parent disc (Disc extrusion)

All patients underwent MRI evaluation with a 0.5 tesla scanning machine. MRI findings analyzed were disc degeneration, extent of disc prolapse, neural foramen compromise, nerve root compression, and miscellaneous findings(ligamentum flavum hypertrophy, facet joint arthritis, canal stenosis). Disc degeneration was graded from 1 to 5 as per Pfirrmann *et al*.^11^ Grade 1–3 were considered insignificant and normal. Grade 4 and 5 were considered as abnormal. Disc herniation was classified as follows; a) Normal: No disc extension beyond the interspace. b) Disc bulge: Circumferential symmetrical disc extension beyond the interspace [Figure 1a]. c) Disc protrusion: Focal or asymmetrical disc extension beyond the interspace with base against the parent disc broader than any other diameter of the protrusion [Figure 1b]. d) Disc extrusion: Focal obvious disc extension beyond the interspace with base against the parent disc narrower than the diameter of the extruding material itself or no connection to parent disc [Figure 1c].

Neural foramen compromise was graded as thecal sac compression, neural foramen compromise, nerve root contact, and nerve root compression.^6^ To avoid inter- and intraobserver variations of MRI findings, all the MRI films were reported by two senior most radiologist (RG) with experience in MRI and who was blinded to the diagnosis. Kappa coefficient was used to assess intra- and interobserver variations. A Kappa value of 0.5 and above was used as a good agreement. Analysis of results was done and clinical findings and MRI findings were correlated to know the association between clinical and MRI findings and significance of MR findings in producing symptoms. Statistical tests (Kappa and Logistic regression analysis) were done to know the association of symptoms with MRI findings.

## RESULTS

123 patients, 59 males and 64 females, who satisfied clinical criteria between June 2003 and January 2008 were initially included in the study. As MRI showed a different diagnosis in four patients, they were excluded from the study. Remaining 119 patients with 290 levels of disc herniation were included in the study.The mean age was 44.83 years (20–72 years). 2 patients were under 20 years of age, 18 patients were in the age group of 21–30, 33 patients were in the age group of 31–40, 30 patients were in the age group of 41–50, 20 patients were in the age group of 51–60, and there were 16 patients above 61 years of age.

### Clinical features

The pain distribution was classified as per the dermatomal level. L5 and S1 dermatomes were involved in 55 and 28 patients, respectively, while 9 patients had nonspecific pain distribution. 4 patients had either L3 or L4 dermatomal distribution of pain, 25 patients had more than one dermatomal level distribution of pain (18 patients had L5 and S1 distribution, while 7 patients had L4 and L5 dermatomal level pain distribution). Unclear multiple level pain distribution was present in two patients. Thus, a total of 80 patients had pain distribution in the L5 dermatomal level (making it the commonest dermotamal level involved). A total of 46 patients had pain distribution in the S1 dermatomal level. 73 patients out of 119 patients had neurological symptoms. Amont this, 36 patients had neurological symptoms in the L5 dermatomal distribution, while 20 patients had S1 dermatomal distribution. 4 patients had L4 level neurological symptoms distribution, and another 13 patients had neurological symptoms involving more than one level (3 patients L4 and L5, 10 patients L5 and S1). 36 patients had neurological deficits. Out of these, 20 patients had both motor and sensory deficits, 13 had only sensory deficits, and 12 had deficits involving more than one level. 2, 16, and 6 patients had a neural deficit of L4, L5, and S1 level, respectively, while 3 patients had L4 and L5 level whereas 7 patients had L5 and S1 level deficits. Two patients had multiple level involvement.

### MRI findings

#### Disc degeneration

90 (75%) patients had disc degeneration of grade 4 and above at two or more levels. Fifty-three (45%) patients had disc degeneration at three or more levels. 287 level disc degenerations (grade 4 and above) were noted in 119 patients (average 2.4).

#### Disc herniation (290 discs level)

Disc bulges were noticed in 208 levels in 119 patients. Disc protrusion was noticed in 56 levels (43 patients). Disc extrusion was noticed in 26 patients with 20 of them having migration of fragment. The Kappa value for interobserver variation was 0.46 and that for intraobserver variation was 0.7. When the Kappa value was calculated for the interobserver variability between a disc bulge and protrusion or extrusion, it was found to be 0.7 while the Kappa value for differentiating between disc protrusion and disc extrusion was 0.39.

#### Neural foramen compromise and nerve root compression

There was evidence of neural foramen compromise at 157 levels out of 290 levels of disc herniation in 119 patients. Nerve root compression was noted in 66 levels. The Kappa value for interobserver variation was 0.6 while that for intraobserver variation was 0.8. Among 208 levels of disc bulges, there was evidence of neural foramen compromise at 139 levels and nerve root compression at 27 levels. Foramen compromise was seen in 12 levels with disc protrusion and 6 levels with disc extrusion. Nerve root compression was noticed in 23 levels with disc protrusion and 16 levels with disc extrusions [Table 1]. Except disc bulges at seven levels, all were reported as diffuse, while those seven levels showed a centrolateral disc bulge. Considering the large number of disc bulges, we did not give importance to this. In disc protrusion and extrusion, the position of disc herniation was noted and its relationship with neural foramina or nerve root was also noted. Out of 56 disc protrusions, 31 were central and 23 were centrolateral while 2 protrusions were far lateral. Out of 26 disc extrusions, 10 were central and remaining 16 were centrolateral.

**Table 1:** Disc herniation and neural canal compromise

Degree of neural canal compromise	Disc bulge	Disc protrusion	Disc extrusion with or without free fragment
No foramen compromise noted (thecal sac compression)	42	21	4
Foramen/lateral recess compromise	139	12	6
Nerve root impingement	27	23	16

#### Miscellaneous findings

A total of 15 patients showed ligament flavum hypertrophy, 34 levels showed stenosis of canal, 19 patients showed facet joint hypertrophy (1 or more levels), and 5 patients had spondylolisthesis.

#### Correlation of results

a) Correlation between the symptomatic level and the MRI level

While correlating clinical and MRI levels of lesion, if multiple level disc prolapses were present, the nerve root compression visible in MRI was used as the MRI level. When only neural foramen compression was seen, the conventional wisdom that L4–5 level produces L5 dermatomal level symptoms and L5–S1 level produces S1 dermatomal level symptoms was used. However, if only one-level neural foramen compression or neural compression was visible in MRI, the same was taken as the MRI level. Kappa statistics were used to correlate the clinical level and the MRI level. If one considers 2-level clinical symptoms as separate levels for statistical analysis, there were total 146 symptomatic levels in 119 patients. The MRI lesion at 25 levels was responsible for symptoms at two levels. Hence, there were 121 MRI levels responsible for symptoms at 146 clinical levels. Except eight levels, all clinical levels of symptoms matched with the MRI level of disc herniation. In addition to this, nine patients had nonspecific distribution of symptoms. The Kappa value for the statistical significance between the clinical level and the MRI level was 0.8. The distribution of the symptomatic MRI disc levels is given in [Table 2].

**Table 2:** Correlation between MRI disc level and dermatomal level

MRI disc level with dermatomal level	No. Patients
L3–4 level producing L3 radiculopathy	2
L3–4 level producing L4 radiculopathy	4
L3–4 level producing L4 and L5 radiculopathy	1
L4–5 level producing L5 radiculopathy	59
L4–5 level producing L4 and L5 radiculopathy	5
L4–5 level producing S1 radiculopathy	4
L4–5 level producing L5 and S1 radiculopathy	3
L5–S1 level producing L5 radiculopathy	9
L5–S1 level producing S1 radiculopathy	34
L5–S1 level producing L5 and S1 radiculopathy	5

Nearly 100% patients with MRI evidence of disc protrusion or extrusion [Table 3] with nerve root compression had symptoms (100% and 96%, respectively) while 70% of patients with a disc bulge and nerve root compression also had symptoms. Disc extrusion with neural foramen compromise also produced symptoms in 83% patients, while foramen compromise in disc bulge and protrusion produced symptoms in some patients (33% and 38%, respectively). When there was no evidence of neural foramen compromise, such levels were mostly asymptomatic despite the type of disc herniation. In fact patients with gross extrusion but with central position and not compromising neural foramen were asymptomatic.

**Table 3:** Correlation between the type of disc herniation and clinical symptoms

Type of herniation	Neural canal compromise	Symptomatic	Asymptomatic	Total	Percentage symptomatic
Disc bulge	Without neural foramen compromise	05	37	42	11.9
	With neural foramen compromise	53	86	139	38.13
	Nerve root compression	19	08	27	70.37
Disc protrusion	Without neural foramen compromise	03	18	21	14.3
	With neural foramen compromise	04	08	12	33.3
	Nerve root compression	22	01	23	96.52
Disc extrusion	Without neural foramen compromise	00	04	04	0
	With neural foramen compromise	05	01	06	83.33
	Nerve root compression	16	00	16	100

The Kappa statistics for the association between the type of disc herniation and symptoms is 0.025 and that for foramen compromise and symptoms is 0.3 indicating that foramen compromise is better associated with symptoms than type of disc herniation that is disc bulge, protrusion, and extrusion.

b) Correlation between the neurological deficit and root compression seen in MRI

36 patients had neurological deficits. Among these, 23 patients had nerve root compression seen in MRI. 21 had disc bulges, 8 had disc protrusion, and 7 had disc extrusion. 18 patients with deficits had disc herniation at three or more levels with neural foramen compromise or nerve root contact. 27 patients had either ligament flavum hypertrophy with stenosis or facet joint hypertrophy at one or more levels. There was evidence of nerve root compression in 66 levels. Among these, 57 were symptomatic with radicular pain while 23 had neurological deficits [Table 4].

**Table 4:** Correlation between the neurological deficit and neural canal compromise seen in MRI

MRI	Symptomatic	Deficit	Without symptoms	Percentage symptomatic
No foramen compromise	8	0	59	11.94
Neural foramen compromise	62	13	95	39.49
Nerve root compression	57	23	9	86.36

c) The position of disc protrusion and extrusion and symptoms

31 disc protrusions were central and 20 of these were asymptomatic; 23 disc protrusions were centrolateral and 20 of these were symptomatic. Two patients had far lateral disc protrusion and were symptomatic with deficits. Among disc extrusions, 10 had central disc extrusion and 5 of these were symptomatic. All those five patients had large extrusions with neural foramen compromise with or without nerve deficits. The remaining five central extrusions were asymptomatic. All 16 centrolateral disc extrusions were symptomatic.

***Association between MRI findings and neurological deficits***

We have tested the association between MRI findings (disc extrusion, multiple level foramen compromise and the presence of miscellaneous findings related to chronicity (facet joint hypertrophy, ligament flavum hypertrophy, and canal stenosis) with neurological deficits, and the presence of multiple level foramen compromise as well as findings related to chronicity show a statistically significant association [Table 5].

**Table 5:** Association between MR findings and neurological deficits

Variables tested	Sensitivity	Positive predictive value	Negative predictive value	Significance
Extrusion and deficit	20	26.9	69.9	0.75
Multiple level compression and deficit	51.4	60	80.9	<0.0001
Findings related to chronicity and deficit	77.1	49.1	69.9	<0.0001

Various MRI findings were analyzed with logistic regression analysis for their association with clinical symptoms and neurological deficits for Odd’s ratio and clinical significance. Tables 6 and 7 give the Odd’s ratio with a 95% confidence interval range and *P* values. Logistic regression analysis for association between MRI findings and clinical symptoms show that there is significant association between evidence of neural foramen compromise seen in MRI and clinical symptoms (Odd’s ratio 6.03, *P*<0.001) as well as between evidence of root compression seen in MRI and clinical symptoms (*P*< 0.001). There was no statistical correlation between clinical symptoms and other MRI findings like disc bulge, disc protrusion and disc extrusion (*P* value 0.013, 0.124 and 0.013 respectively). Similary logistic regression analysis for the association between neurological deficits and MRI findings show better significance between presence of multiple level foramen compromise seen in MRI and neurological deficits (*P* value 0.05) compared to other MRI findings [Table 7].

**Table 6:** Association between clinical symptoms and MRI findings

MRI findings	Odd’s ration	95% CI interval	*P* value
Disc bulge	0.203	0.058–0.711	0.013
Disc protrusion	0.341	0.087–1.343	0.124
Disc extrusion	4.914	1.406–17.175	0.013
Neural foramen compromise	6.032	2.541–14.318	<0.001
Root compression	41.756	14.810–117.732	<0.001

**Table 7:** Association between the neurological deficit and MRI findings

MRI findings	Odd’s ratio	95% CI interval	*P* value
Disc protrusion	0.639	0.191–2.140	0.468
Disc extrusion	1.123	0.347–3.636	0.847
Root compression	2.543	0.945–6.845	0.06
Multiple level foramen compromise	2.693	0.966–7.506	0.05
Findings of chronicity	2.363	0.840–6.647	0.1

## DISCUSSION

Very few studies have correlated clinical findings with MRI findings^3^,^5^,^8^,^9^ These studies also gave contrasting reports and were inconclusive. Milette *et al*.^5^ found that the loss of disc height or abnormal signal intensity is highly predictive of symptomatic tears. Presence of a disc bulge or protrusion does not have additional significance. Beattie *et al*.^8^ found that the presence of disc extrusion and severe nerve root compression at one or multiple sites is strongly associated with distal leg pain. However, Rankine *et al*.^9^ in their study opined that pain drawing is not a good predictor of nerve compression on MRI with a poor correlation. Borenstein and others^3^ clearly opined that MRI findings were not predictive of the development or duration of low back pain and that clinical correlation is essential. This particular study was done on asymptomatic individuals.

We evaluated the correlation between clinical and MRI findings. In most cases, pain distribution could be correlated with a particular level making it easy to compare the clinical and MRI levels. Our results show that there is a very good correlation between the clinical level and MRI level. The Kappa coefficient for agreement was 0.8 which is very high. Only nine clinical levels did not correlate with the MRI level. However, when closely analyzed, although we have found that the clinical level and the MRI level with evidence of nerve root compression were matching, the MRI level may not be the same as the conventional wisdom of L4–5 causing L5 radiculopathy and so on. In our study the L4–5 disc prolapse was not only responsible for L5 radiculopathy in 67 (94.36%) cases, but also responsible for L4 radiculopathy in 5 (7%) cases and S1 radiculopathy in 7 (9.8%) cases. Similarly, L5–S1 radiculopathy was responsible for S1 radiculopathy in 39 (81.25%) cases, while L5 radiculopathy in 14 (29.16%) cases. These findings clearly emphasize the need for accurately assessing at which level neural foramen compromise and nerve root compression are present before considering surgical options. So, while the level of disc prolapse correlates well with the clinical level and MRI may not be essential for clinical diagnosis, MRI is definitely essential when surgery is planned.

Also, not all MRI lesions have symptoms. In our study, out of 169 levels of disc lesions only 89 are symptomatic. MRI is a very sensitive test for identifying disc lesions but not specific.

Which of MRI findings are likely to produce symptoms is an important issue. Those MRI findings with neural foramen compromise and nerve root compression are likely to be more symptomatic than those without neural foramen compromise. The position of disc herniation can affect the neural foramen compromise as central protrusion and extrusions are less likely to cause neural foramen compromise while centrolateral or far lateral lesions in all cases produced neural foramen compromise. This means that a patient with a disc bulge with neural foramen compromise is more likely to have symptoms than a patient with disc extrusion even if it is central and does not produce neural foramen compromise. In few studies, it has been shown that disc extrusion can have poor prognosis and high disability.^11^,^12^ In our study, though the percentage of patients with disc extrusion having foramen compromise or root compression is higher compared to those with a disc bulge producing foramen compromise, a pure central disc extrusion or protrusion is asymptomatic in most cases. From our study we can see that a centrolateral disc extrusion, centrolateral disc protrusion, and disc bulge with neural foramen compromise are more likely to cause symptoms in that order while central disc protrusions and extrusions as well as disc bulges without foramen compromise are less likely to produce symptoms. These findings are also important when surgical options are considered. For example, a patient may have a double-level disc prolapse, one with a central protrusion and one with a disc bulge with neural foramen compromise. In such cases, one with a disc bulge and foramen compromise is likely to cause symptoms and after clinical correlation gives a clue for a surgical approach.

Neurological deficit is well correlated with nerve root compression in our study. However, all root compressions seen in MRI need not have neurological deficits. Instead we found that patients with multiple level foramen compromise have neurological deficits. This indicates that there is a possibility of multiple level pressures on nerve roots in these cases. A single-level neural compression may not be sufficient to produce neural deficits unless it is very severe. However, when a nerve root compression is visible on MRI, it is more likely to produce symptoms as in 86.36% (57 out of 66 patients) patients in our study. When only neural foramen compromise was seen, 39.49% [Table 3] patients were symptomatic. In those without neural foramen compromise, only 11.94% [Table 3] patients were symptomatic. This clearly shows that MRI evidence of nerve root compression, although not specific of neurological deficits, is more likely to produce symptoms.

Since 0.5 T MRI scanning machine was used, the identification of nerve root compression was problematic, and this was a limitation in our study. At some levels it was difficult to see the nerve root and hence the case was reported as neural foramen compromise. This could be the reason (in a small number of patients) for neurological deficits without nerve compression seen in MRI. Also it was very difficult to differentiate between the root contact and root compression which showed very high inter- and intraobserver variations and hence we merged root contact and compression together to include them as root compression in our classification.

MRI findings like facet joint hypertrophy and ligament flavum hypertrophy with stenosis of canal were more commonly seen in patients with neurological deficits. Out of 36 patients with neurological deficits, 12 had ligamentum flavum hypertrophy at one or more levels, 6 had facet joint hypertrophy, and 22 had stenosis. In contrast, there was evidence of ligamentum flavum hypertrophy in 11 patients, and lumbar canal stenosis in 15 patients in the remaining 83 patients without a neurological deficit. Logistic regression analysis shows a positive association between these findings and the neurological deficit.

The disc extrusion can be strongly associated with pain.^11^,^12^ However, in our study we found that it is not the disc extrusion that is more symptomatic, and the position of disc extrusion is more predictive of symptoms. Disc protrusions and extrusions can be associated with neural foramen compromise in most cases due to their focal herniation and that may be the reason why disc extrusions can be more likely to be symptomatic with distal leg pain. Beattie and others,^8^ have also found that root compression can be associated with a high degree of distal leg pain which is true in our study also.

There are a few studies correlating the morphology of disc herniation with symptoms. Carragee and others^13^ found that herniated disc morphology and the size of disc have a bearing on the surgical outcome and not on patients treated conservatively. A high-resolution MRI scan is essential to accurately assess the size of the herniated fragment. Presently disc herniations are classified as small and large and are totally unreliable. It is suggested to accurately measure the size of the herniated fragment and assess its correlation with clinical symptoms and prognostic values.

## CONCLUSIONS

Clinical findings correlate well with MRI findings, but all MRI abnormalities need not have a clinical significance. The presence of centrolateral disc protrusion and extrusions with gross neural foramen compromise is invariably associated with clinical signs and symptoms. Disc bulges with thecal sac compromise or central protrusions and extrusions without significant neural foramen compromise are clinically insignificant. The presence of neural foramen compromise is more important in determining the clinical signs and symptoms while the type of disc herniation (bulge, protrusion, or extrusion) correlates poorly with clinical signs and symptoms. Whenever there are multiple level disc lesions with neural foramen compromise, patients are likely to have objective neurological deficits.
